# Independent Predictors of Mortality in Systemic Sclerosis-Associated Pulmonary Arterial Hypertension: A Systematic Review and Meta-Analysis

**DOI:** 10.7759/cureus.39730

**Published:** 2023-05-30

**Authors:** Zineb Barkhane, FNU Nimerta, Sualeha Zulfiqar, Saleha Dar, Muhammad Sohaib Afzal, Amna Zaree, Rahul Adwani, Sujith K Palleti

**Affiliations:** 1 Department of Neurology, Université Hassan II de Casablanca, Casablanca, MAR; 2 Department of Medicine, Jinnah Sindh Medical University, Karachi, PAK; 3 Department of Internal Medicine, Rawalpindi Medical University, Rawalpindi, PAK; 4 Department of Medicine, Louisiana State University Health Sciences Center, Shreveport, USA; 5 Department of Medicine, Shalamar Medical and Dental College, Lahore, PAK; 6 Department of Medicine, Dow University of Health Sciences, Karachi, PAK; 7 Department of Nephrology, Edward Hines Jr. Veterans Administration Hospital, Hines, USA; 8 Department of Nephrology, Loyola University Medical Center, Maywood, USA

**Keywords:** meta-analysis, systemic sclerosis, pulmonary arterial hypertension, mortality, predictors

## Abstract

The aim of this study is to determine the predictors of mortality in patients with systemic sclerosis-induced pulmonary arterial hypertension (SSc-PAH). This systematic review and meta-analysis were carried out according to the Preferred Reporting Items for Systematic Reviews and Meta-analyses (PRISMA) Statement guidelines. We searched the PubMed, EMBASE, and Web of Science databases from January 2010 to April 2023 using the following keywords: "systemic sclerosis," "pulmonary arterial hypertension," "death," and "predictors," along with medical subject headings (MeSH), to identify relevant studies. A total of eight studies with a total of 530 patients were included in the present systematic review and meta-analysis. The pooled one-year, three-year, and five-year survival was 90% (95% CI: 86-93%), 66% (95% CI: 59-72%), and 44% (95% CI: 23-65%), respectively. Factors associated with mortality in SSc-PAH included age (p-value: 0.02), male gender (p-value: 0.008), pericardial effusion (p-value: 0.003), cardiac index (p-value: 0.0001), six-minute walking distance (p-value: 0.04), pulmonary arterial pressure (PAP) (p-value: 0.01), and New York Heart Association (NYHA) classification (p-value: 0.0002). The findings of this study have important clinical implications. Assessing and managing the identified predictors, such as age, gender, pericardial effusion, PAP, cardiac index, and NYHA class, could help identify individuals at higher risk of mortality and guide treatment strategies.

## Introduction and background

Systemic sclerosis (SSc) is a chronic autoimmune disease that affects multiple systems in the body. It is characterized by abnormalities in blood vessels, tissue scarring (fibrosis), and immune system activation. SSc can be classified into two types based on the degree of skin involvement: limited cutaneous SSc or diffuse cutaneous SSc [1]. SSc presents with various systemic manifestations, including key features such as swollen fingers or thickened skin. It can also affect the muscles and joints, causing stiffness and a limited range of motion. Other common complications involve the lungs (interstitial lung disease), the gastrointestinal system (impaired motility), and the heart (cardiac involvement) [1]. SSc is responsible for a significant number of cases of pulmonary arterial hypertension (PAH), which is observed in approximately 10-15% of SSc patients [2-3].

Individuals with SSc-related PAH (SSc-PAH) generally have a less favorable prognosis compared to those with idiopathic PAH (iPAH) [4-5]. PAH is a leading cause of mortality in SSc, contributing to a higher risk of death in affected individuals [6]. The initial clinical signs of SSc-PAH are generally vague and not specific to the condition. Common symptoms include shortness of breath, fatigue, and difficulty tolerating physical activity. In the past, a significant number of SSc-PAH patients were diagnosed at an advanced stage, with the identification of the condition often delayed for more than two years after the onset of symptoms [7]. This delay in diagnosis can be attributed to the nonspecific nature of the initial symptoms. It is widely acknowledged that individuals with milder disease at the time of diagnosis have a higher survival rate, both in the case of SSc and iPAH [8].

Although there have been advancements in therapies that have improved survival rates, individuals with SSc-PAH continue to have lower survival rates compared to those with iPAH and connective tissue disease-associated PAH [9]. The one-year, two-year, and three-year survival rates for SSc-PAH are reported as 90%, 78%, and 56%, respectively. In contrast, the corresponding survival rates for iPAH are 92%, 75%, and 66%, respectively [10]. It should be noted that these figures are based on the incident and prevalent SSc-PAH cohort data, and as a result, there may be a survival bias, meaning that the actual survival rates for newly diagnosed cases of SSc-PAH may be lower than these reported figures.

Several studies have assessed the risk factors affecting mortality in SSc-PAH. It is vital to identify such factors to ensure optimal care and facilitate appropriate monitoring. However, there is no consensus regarding the factors affecting mortality in SSc-PAH. Therefore, a systematic review and meta-analysis are needed to understand the different factors associated with mortality in SSc-PAH. The aim of this systematic review and meta-analysis is to determine the predictors of mortality in patients with SSc-PAH.

## Review

Methodology

This systematic review and meta-analysis were carried out according to the Preferred Reporting Items for Systematic Reviews and Meta-analyses (PRISMA) Statement guidelines.

Search Strategy and Study Selection

We searched the PubMed, EMBASE, and Web of Science databases from January 2010 to April 2023 using the following keywords along with their synonyms: "systemic sclerosis," "pulmonary arterial hypertension," "death," and "predictors," along with medical subject headings (MeSH), to identify relevant studies. Additionally, the reference lists of all included articles were manually searched to identify additional relevant records. All records were manually screened independently by two authors. Initially, screening was done using titles and abstracts. Full texts of all eligible records were obtained, and a detailed assessment was conducted based on pre-defined inclusion and exclusion criteria. Studies were included if they fulfilled the following criteria: (a) the study included individuals with a diagnosis of SSc-associated PAH; (b) assessed predictors of mortality; and (c) included adult patients. We excluded reviews, case reports, case series, and studies published in languages other than English. Any disagreements in the study search and selection process were resolved through discussion.

Data Extraction and Statistical Analysis

Data from the included studies were independently extracted by two authors using a pre-designed standardized data collection form. The collected data from the included studies included author name, year of publication, region, sample size, and data on factors (including demographic and clinical factors). To assess the relationship of each factor associated with mortality in patients with SSc-associated PAH, hazard ratios (HR) were calculated along with 95% confidence intervals (CI). A p-value less than 0.05 was considered significant. Heterogeneity was determined using I-square statistics, with an I-square value of more than 50% considered significant for heterogeneity, and a random-effects model was used. Otherwise, a fixed-effect model was used. Data analysis was performed using RevMan Version 5.4.1 (The Cochrane Collaboration, London, United Kingdom) and STATA Version 16.0

Results

**Figure 1:**
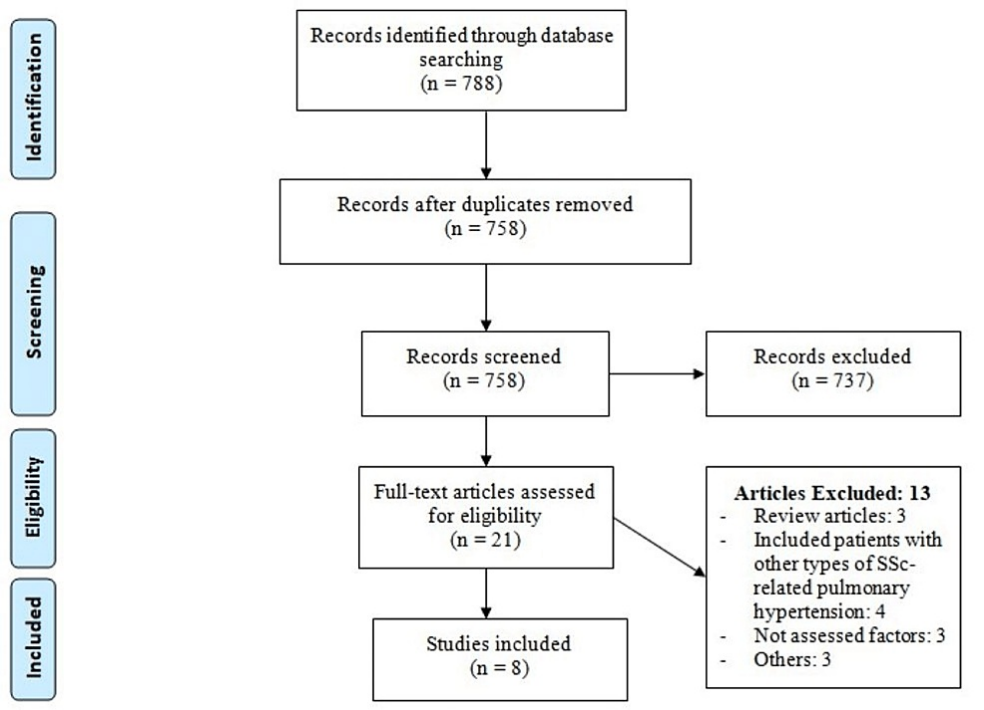
PRISMA flowchart of study selection

Our search of online databases from January 2010 to April 2023 retrieved 788 citations. After the abstracts and titles were evaluated, 737 studies did not fulfill the inclusion criteria. The full text of the remaining 21 studies was evaluated. Finally, eight studies with a total of 530 patients were included in the present systematic review and meta-analysis. Figure 1 shows the PRISMA flowchart of study selection. Table 1 shows the characteristics of included studies. All the selected studies were observational studies. The pooled one-year, three-year, and five-year survival was 90% (95% CI: 86-93%), 66% (95% CI: 59-72%), and 44% (95% CI: 23-65%) respectively.

**Table 1:** Characteristics of included studies

Author Name	Publication Year	Region	Multicenter	Study Duration (Years)	Number of Patients	Age (Years)	Male (%)
Campo et al. [11]	2010	United States	No	5	76	60.7	15.8
Chung et al. [12]	2014	United States	Yes	3	128	60.4	16
Chung et al. [13]	2014	United States	Yes	3	500	61.6	12.6
Kolstad et al. [14]	2018	United States	Yes	8	160	60.1	10.7
Launay et al. [15]	2010	France	No	3	49	59	26.5
Launay et al. [16]	2013	France	Yes	3	85	64.9	18
Mathai et al. [17]	2010	United States	No	3	55	57	11
Morrisroe et al. [18]	2017	United States	No	5	132	62.3	15

Predictors of mortality

Age

**Figure 2:**
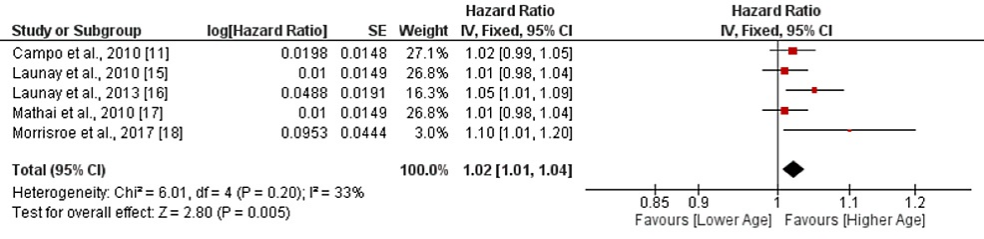
Effect of age on mortality. HR>1 shows the risk of mortality increased with increased age, while HR<1 shows the risk of mortality decreased with increased age Sources: [11,15-18]

Five studies were included in the pooled analysis of the effect of age on mortality. The pooled analysis showed that an increase in age is associated with a greater risk of mortality (HR: 1.02, 95% CI: 1.01-1.04, p-value: 0.02), as shown in Figure 2. No significant heterogeneity was reported among the study results (I-square: 33%). All studies reported that with increasing age, the risk of mortality also increased. However, only one study reported a significant result [16]. Chung et al. [12] reported that patients above the age of 60 had a threefold increased risk of death (HR: 3.0, 95% CI: 1.1-8.4, p-value: 0.22). Table 2 shows the quality assessment of the included studies.

**Table 2:** Quality assessment of included studies

Author Names	Selection	Comparison	Outcome	Overall
Campo et al. [11]	3	2	3	Good
Chung et al. [12]	4	2	2	Good
Chung et al. [13]	3	2	3	Good
Kolstad et al. [14]	3	1	2	Fair
Launay et al. [15]	2	2	3	Good
Launay et al. [16]	3	1	2	Fair
Mathai et al. [17]	4	2	2	Good
Morrisroe et al. [18]	3	2	3	Good

Gender

**Figure 3:**
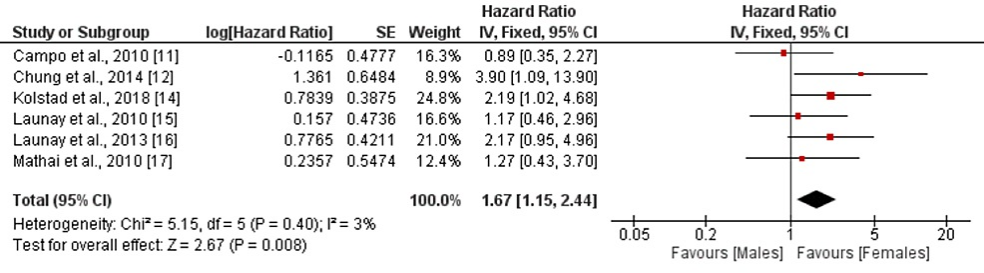
Effect of gender. HR>1 shows a higher risk of mortality in males, and HR<1 shows a higher risk of mortality in females Sources: [11-12,14-17]

A total of six studies were included in the analysis, examining the risk of mortality as an outcome in relation to gender as the independent factor. When compared to the reference category of female, the HR for mortality in the male gender was 1.67 (95% CI: 1.15-2.44) with a statistically significant p-value of 0.008 as shown in Figure 3. There was minimal heterogeneity observed among the study results, as indicated by an I-square value of 3%. Five studies reported higher mortality risk among males, but only two reported significant differences [12,14].

Pericardial Effusion

**Figure 4:**
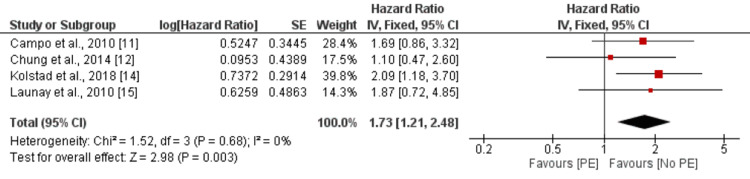
Effect of pericardial effusion. HR>1 shows a higher risk of mortality in patients with pericardial effusion, and HR<1 shows a higher risk of mortality in patients without pericardial effusion PE: pericardial effusion Sources: [11-12,14-15]

The risk of mortality was assessed as the outcome variable, with pericardial effusion serving as the independent factor. The reference category for comparison was no pericardial effusion. The pooled analysis of four studies revealed that the presence of pericardial effusion was associated with a higher risk of mortality, as indicated by an HR of 1.73 (95% CI: 1.21-2.48). This association was statistically significant, with a p-value of 0.003 as shown in Figure 4. Furthermore, there was no significant heterogeneity observed among the study results, as indicated by an I-square value of 0%.

Cardiac Index

**Figure 5:**
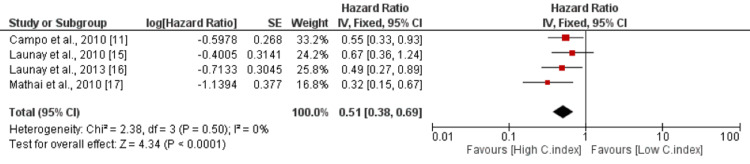
Effect of cardiac index. HR>1 shows a risk of mortality increased with increased cardiac index, and HR<1 shows the risk of mortality decreased with increased cardiac index C.index: cardiac Index Sources: [11,15-17]

The risk of mortality was examined as the outcome variable, with the cardiac index as the independent factor. The cardiac index was treated as a continuous variable in the analysis. The pooled results from four studies indicated that a higher cardiac index was associated with a significantly reduced risk of mortality, with an HR of 0.51 (95% CI: 0.38-0.69). The association was highly significant, as evidenced by a p-value of 0.0001 as shown in Figure 5. Furthermore, no significant heterogeneity was observed among the study results, with an I-square value of 0%. These findings suggest that a higher cardiac index is associated with a lower risk of mortality.

Six-Minute Walking Distance

**Figure 6:**
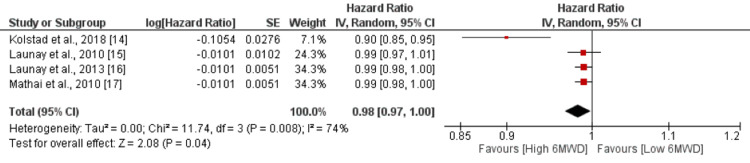
Effect of 6MWD. HR>1 shows risk of mortality increased with 6MWD, and HR<1 shows risk of mortality decreased with increased 6MWD 6MWD: six-minute walking distance Sources: [14-17]

The risk of mortality was assessed as the outcome, with the six-minute walking distance considered as the independent factor. The six-minute walking distance was treated as a continuous variable in the analysis. The pooled results from four studies showed that there was a slight, but statistically significant, association between the six-minute walking distance and the risk of mortality. The HR was 0.98 (95% CI: 0.97-1.00), indicating a slightly reduced risk of mortality with a higher walking distance. The p-value was 0.04, suggesting statistical significance as shown in Figure 6. However, there was moderate heterogeneity among the study results, as indicated by an I-square value of 74%. Chung et al. [13] reported a significantly higher risk of mortality in patients having a six-minute walking distance of less than 165 meters compared to their counterparts.

Pulmonary Arterial Pressure

**Figure 7:**
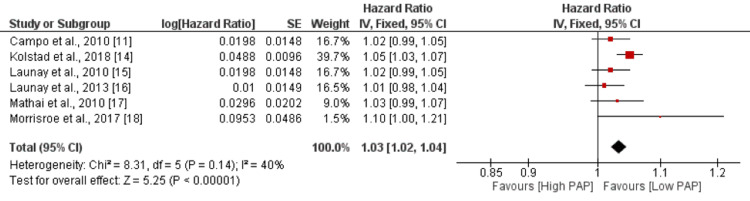
Effect of PAP. HR>1 shows risk of mortality increased with increased PAP, and HR<1 shows risk of mortality decreased with increased PAP PAP: pulmonary arterial pressure Sources: [11,14-18]

The pooled analysis of six studies revealed that an increase in pulmonary arterial pressure (PAP) was associated with a slightly higher risk of mortality, with an HR of 1.03 (95% CI: 1.02-1.04). The association was statistically significant, as indicated by a p-value of 0.01 as shown in Figure 7. However, no significant heterogeneity was reported among the study results, with an I-square value of 40%.

Pulmonary Vascular Resistance

**Figure 8:**
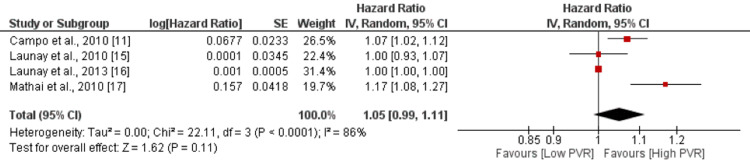
Effect of PVR HR>1 shows risk of mortality increased with increased PVR, and HR<1 shows risk of mortality decreased with increased PVR PVR: pulmonary vascular resistance Sources: [11,15-17]

The pooled analysis of four studies indicated a slight increase in the risk of mortality with higher pulmonary vascular resistance (PVR) values, as reflected by an HR of 1.05 (95% CI: 0.99-1.11). However, the association did not reach statistical significance, as the p-value was 0.11 as shown in Figure 8. Notably, there was a high degree of heterogeneity among the study results, with an I-square value of 86%.

New York Heart Association Classification

**Figure 9:**
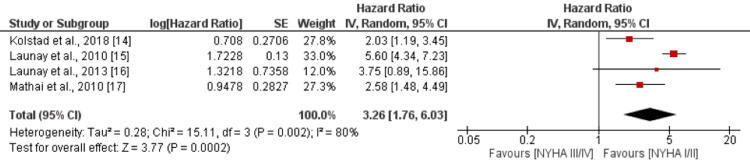
Effect of NYHA classification. HR>1 shows a high risk of mortality in NYHA III/IV, and HR<1 shows a high risk of mortality in NYHA I/II NYHA: New York Heart Association Sources: [14-17]

The pooled analysis of four studies revealed a strong association between higher New York Heart Association (NYHA) classification and an elevated risk of mortality. The HR was 3.26 (95% CI: 1.76-6.03), indicating a substantially increased risk. The association was highly significant, as evidenced by a p-value of 0.0002 as shown in Figure 9. However, there was a high level of heterogeneity observed among the study results, with an I-square value of 80%. Chung et al. reported that individuals with class 3 and class 4 of the NYHA classification are at an increased risk of mortality compared to their counterparts [13].

Discussion

In this meta-analysis, we explored the mortality rate in SSc-PAH and the associated predictors. The pooled one-year, three-year, and five-year survival rates were 90% (95% CI: 86-93%), 66% (95% CI: 59-72%), and 44% (95% CI: 23-65%), respectively.

Age was found to be a significant predictor of mortality in the present meta-analysis. Increased age is associated with a higher risk of mortality in patients with SSc-associated pulmonary hypertension (PH). The pooled analysis also reported a higher risk of death in males compared to women. Chung et al. [13] reported that men over the age of 60 years are at a greater risk of death compared to their counterparts. Out of the six included studies that assessed the effect of gender on mortality, five studies reported a higher incidence in males [12,14-17]. Two out of the five studies reported significant differences in mortality between males and females [12,14]. These results are consistent with the study conducted by Condliffe et al., who reported that female sex and younger age were protective in patients with SSc-PAH [8]. The study conducted by Pasarikovski et al. reported that males had a shorter time to PAH diagnosis from SSc diagnosis, with the average time to SSc-PAH diagnosis being more than three years earlier in males compared to females [19]. In conjunction with our findings, this could imply that while PAH is less common among males, they may experience more severe outcomes.

In the study conducted by Lefevre et al. [20], a correlation was found between overall survival and PVR in patients with SSc-associated PH (SSC-PH). This means that higher PVR levels are associated with poorer survival outcomes in these patients. Moreover, the current meta-analysis supports these findings by demonstrating that higher PVR levels significantly increase the risk of death in patients with SSc-PAH. Chung et al. conducted a study specifically investigating the impact of PVR on mortality in patients with SSc-PAH. They found that patients with a PVR greater than 32 Wood units had a significantly higher risk of mortality compared to their counterparts with lower PVR levels. In fact, the risk of mortality in patients with PVR > 32 Wood units was reported to be 12.47 times higher than in those with lower PVR levels [13].

The meta-analysis also revealed that a higher cardiac index, which measures the efficiency of the heart's pumping function, is associated with a reduced risk of mortality in these patients. This suggests that maintaining a higher cardiac index can potentially prevent death in individuals with SSc-PAH. Studies have shown that a higher cardiac index is associated with better survival outcomes in SSc-PAH patients. For instance, in a systematic review and meta-analysis by Hachulla et al., a higher cardiac index was found to be a positive prognostic factor in SSc-PAH patients [21]. By maintaining a high cardiac index, the heart can effectively compensate for the hemodynamic impairments associated with SSc-PH, reducing the risk of mortality. These findings emphasize the importance of assessing and managing PVR and cardiac index in patients with SSc-PAH. Lowering PVR levels and maintaining a higher cardiac index can potentially improve survival outcomes in these patients. Further research and clinical interventions focused on optimizing these factors may be warranted to improve the prognosis of individuals with SSc-PH [22].

Our study revealed that the six-minute walk distance also served as a factor, contrary to expectations. Traditionally, this test is considered inadequate for evaluating the severity of PH in patients with SSc [23-24]. The limitations of using the six-minute walk distance in SSc have been widely recognized. It is highly likely that this test encompasses various factors not directly associated with PAH, such as musculoskeletal impairments or depression [25-26]. Consequently, these findings suggest that the six-minute walk distance cannot be definitively regarded as an efficient prognostic factor in SSc-PAH. However, our analysis clearly demonstrates that the six-minute walk distance does serve as a prognostic factor in SSc patients with PAH and includes a larger number of patients through pooling studies. This outcome underscores the importance of exercising caution when interpreting data from smaller studies in SSc.

The findings suggest that patients with pericardial effusion are at an elevated risk of mortality due to systemic-induced PAH. This implies that the presence of pericardial effusion may be associated with more severe forms of PAH in SSc patients, leading to worse outcomes and an increased risk of death. The exact mechanisms underlying this association are not fully understood and may require further research. However, it is plausible that pericardial effusion contributes to the development or progression of PAH through various factors such as increased right-sided heart pressures, impaired diastolic function, or other related pathophysiological processes [27].

The current meta-analysis showed an association between higher NYHA classes and the risk of death in SSc-PAH. The NYHA class is a grading system used to assess the functional capacity and symptom severity in patients with heart failure or PAH. The increased risk of death in patients with a higher NYHA class may be attributed to factors such as more advanced disease progression, greater hemodynamic impairment, reduced functional capacity, and increased susceptibility to complications. Additionally, patients with higher NYHA classes often require more intensive treatment and may have poorer responses to therapy [28].

Study Limitations

Our meta-analysis has certain limitations. Some factors were evaluated in less number of studies, therefore, weakening the role of meta-analysis. Secondly, the number of studies included in this meta-analysis and the number of patients included in each study was low, therefore, reducing the power of the results. Studies were conducted in a few countries only. Therefore, the generalizability of these studies is lower. In the future, more studies are required with a larger sample size and in various regions to evaluate the risk factors of mortality in SSc-PAH.

## Conclusions

In conclusion, this systematic review and meta-analysis investigated the mortality rate and predictors of mortality in SSc-PAH. The pooled analysis of eight studies with a total of 530 patients revealed that the one-year, three-year, and five-year survival rates were 90%, 66%, and 44%, respectively. The study identified that increased age, male gender, pericardial effusion, lower cardiac index, higher PAP, and NYHA class are significant predictors of mortality in SSC-PAH patients. The findings of this study have important clinical implications. Assessing and managing the identified predictors, such as age, gender, pericardial effusion, pulmonary arterial pressure, cardiac index, and NYHA class, could help identify individuals at higher risk of mortality and guide treatment strategies. Strategies aimed at reducing pulmonary arterial pressure, optimizing cardiac index, and addressing pericardial effusion may improve survival outcomes in SSc-PAH patients.
